# A multi-assay assessment of insecticide resistance in *Culex pipiens* (Diptera: Culicidae) informs a decision-making framework

**DOI:** 10.1371/journal.pone.0324194

**Published:** 2025-06-09

**Authors:** Kristina Lopez, Patrick Irwin, Daniel Bartlett, Christopher Kukla, Susan Paskewitz, Lyric Bartholomay

**Affiliations:** 1 Department of Entomology, University of Wisconsin – Madison, Madison, Wisconsin, United States of America; 2 North Shore Mosquito Abatement District, Northfield, Illinois, United States of America; 3 Northwest Mosquito Abatement District, Wheeling, Illinois, United States of America; 4 Impact Networking, Lake Forest, Illinois, United States of America; 5 Department of Pathobiological Sciences, University of Wisconsin – Madison, Madison, Wisconsin, United States of America; Benha University Faculty of Veterinary Medicine, EGYPT

## Abstract

Insecticide resistance (IR) is an increasing problem globally, making control of vector-borne diseases more difficult. Reduced susceptibility to permethrin in *Culex pipiens*, an important vector for West Nile virus, has been reported across the US based on a standardized laboratory method: the CDC bottle bioassay. This bioassay uses a rapid phenotypic outcome to reveal evidence for IR, but how this translates to the effectiveness of formulated products used in an operational setting is unclear. Therefore, other methods for IR monitoring are recommended to quantify IR or evaluate formulated products against field populations in real-world conditions. To compare some of the available methods, we collected populations of *Cx. pipiens* from six sites in the Northwest Mosquito Abatement District (Cook Co., Illinois), and used a susceptible laboratory strain of *Cx. pipiens* as a control, to test for IR to pyrethroids using CDC bottle bioassays, caged field trials, and topical applications. CDC bottle bioassays suggested that *Cx. pipiens* from this area exhibit IR to both etofenprox and Sumithrin®. Caged field trials with ultra-low volume Anvil® 10 + 10 (Sumithrin®) demonstrated resistance to the product and underscored the need for inclusion of a susceptible control to differentiate IR from inadequate product distribution. Topical applications revealed low to high levels of resistance to synergized and unsynergized pyrethroids (etofenprox, Sumithrin®, and deltamethrin) in all field populations. Based on these data, we provide a new decision-making tree for mosquito control professionals which will guide selection of the most optimal assay for IR surveillance based on their goals, needs, and resources.

## Introduction

Globalization and climate change have contributed to the expansion and proliferation of mosquitoes and the disease agents they carry [1]. Chemical control of adult mosquitoes with insecticides, i.e., adulticide, is the only practical way for a mosquito control organization (MCO) to rapidly reduce arbovirus transmission risk [2]. Ground and aerial adulticide applications, typically with pyrethroid-based products, are key practices during arboviral disease outbreaks. However, multiple reports suggest that unrecognized insecticide resistance (IR) in mosquito populations could undermine successful adult mosquito control [3,4]. In an effort to combat and better understand the risk of IR in the context of public health entomology, monitoring for insecticide efficacy and resistance is a core tactic in an integrated mosquito management program [2]. Other core tactics of an integrated mosquito management program include community engagement, disease and mosquito surveillance, mapping, setting action thresholds, biological control, larval source reduction, and targeted applications of larvicides and adulticides [2].

The city and suburbs of Chicago, IL, USA are a hot spot for West Nile virus (WNV) [5,6]. In this region, MCOs and community decision makers rely on adulticide treatment during periods of high WNV activity to reduce risk of WNV transmission by *Culex* spp. vectors [7]. Previous efforts to evaluate the effectiveness of adulticides in this region have produced varying results, as even when significant reduction was observed, impacts on the mosquito population were short-lived and minimal [6–10]. These differences could be attributed to physical limitations like inadequate drift of the product due to inconsistent wind, varying levels of shelter, and objects blocking the spray cloud. Additional biological processes that may contribute to these results include mismatched application and egression times, rapid population rebound, differential impacts on subgroups of adult mosquito populations [7,10], species-based sensitivity to the insecticide, and IR. Determining the magnitude and distribution of IR in this area is a critical step for predicting the outcomes of mosquito control efforts and aid in decision-making in the interest of controlling WNV vectors. Towards this goal, the CDC bottle bioassay is the first-line in the U.S. for assessing the presence of IR traits in a mosquito population, as recommended by the American Mosquito Control Association [2]. In the Chicago area, IR has been detected in many *Culex pipiens* L. populations based on CDC bottle bioassay results [4,11].

The CDC bottle bioassay is a time-to-mortality based assay with specific insecticide doses and diagnostic times based on the target mosquito species; the resulting data are used for comparisons across regions and time periods [12]. Indeed, increased usage of the CDC bottle bioassay is revealing widespread IR across the U.S. [3,4] and the results clearly show that IR is a rapidly developing problem. By design, the CDC bottle bioassay alerts the user to IR only to the active ingredient in adulticidal products, but due to specific dosage and method of exposure, cannot provide indication of the strength of resistance or the potential of ultra-low volume (ULV) application failure. Results from this bioassay cannot directly be used for making operational ULV application decisions as it does not account for sublethal/delayed effects or formulated products, which often contain inert ingredients and synergists to enhance toxicity. Therefore, in order to make operational decisions about how to use resources at hand or pivot to new products, additional IR assays must be done.

Other methods of evaluating insecticide efficacy, including caged field trials (CFTs) and topical applications, supplement CDC bottle bioassay data and provide information about formulated product effectiveness and quantitative understanding of the level of IR in a population. CFTs utilize cages of mosquitoes positioned down-wind of a ULV sprayer to determine if the formulated product will kill mosquitoes in field conditions. This assay is very informative in operations as it most closely represents a real ULV adulticide application, but is subject to spurious results because a lack of mortality may be due to insufficient adulticide contact or IR. Topical applications of insecticide are widely used in insecticide discovery and product development, as toxicity can be measured and compared very precisely. This assay requires more effort, but can be highly tailored in dosage, insecticide, synergist, and mosquito species. Additionally, droplet deposition on the thorax is more representative of real-world exposure through an adulticide cloud [13–15] and yields higher toxicity than tarsal contact [16,17].

To better understand insecticide efficacy and resistance in field-collected mosquitoes from the greater Chicago area, and to determine which assay is most informative, we used the CDC bottle bioassay, CFTs, and topical application to evaluate susceptibility in local *Cx. pipiens* from the Northwest Mosquito Abatement District (NWMAD) to etofenprox, Sumithrin®, and deltamethrin. Based on this comprehensive investigation, we provide a guide for assessments of insecticide efficacy and resistance in field-collected mosquitoes and making informed decisions about best practices.

## Materials and methods

### Mosquito collection & rearing

**Fig 1:**
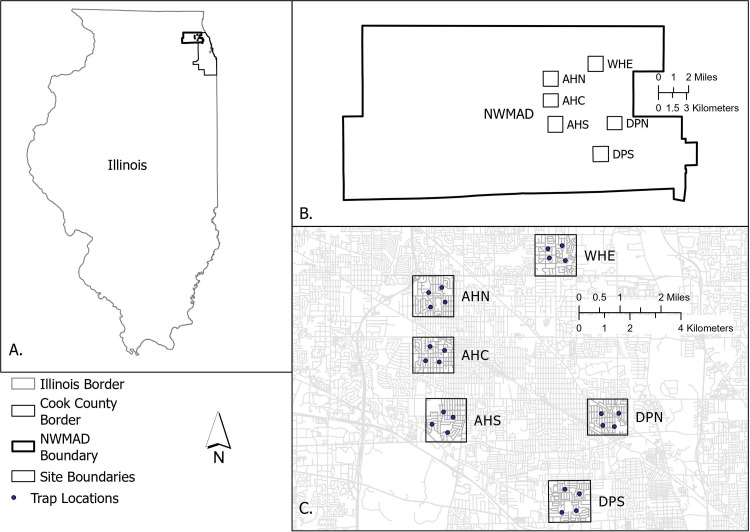
Map of study sites. NWMAD: Northwest Mosquito Abatement District; AHN: Arlington Heights North; AHC: Arlington Heights Central; AHN: Arlington Heights South; WHE: Wheeling; DPN: Des Plaines North; DPS Des Plaines South.

*Cx. pipiens* egg rafts were collected from six study sites within the boundaries of the NWMAD (Fig 1). Within each site, eggs were collected from CDC gravid trap bins (Model 1712, John W. Hock Company, Gainesville, FL) baited with an hay infusion previously described [10] at four residential properties (Fig 1). Collections occurred in July and August of each year (epidemiological weeks 27–35). Egg rafts were placed into separate cups prior to hatching, then identified at second instar [18]. *Cx. pipiens* larvae from the same site were pooled together and other species were discarded. Rafts from a susceptible lab strain of *Cx. pipiens* were obtained from the University of Wisconsin – Madison and reared in the same conditions. Larvae were fed ground TetraMin® tropical fish flakes (Spectrum Pet Brands LLC, Blacksburg, VA) and maintained at a 16:8 hour light dark cycle at 27°C and 80% RH. Adult mosquitoes were fed a 10% sucrose solution. For all experiments, F_0_ adults from field-collected eggs were used in pursuit of true ‘wild-type’ mosquitoes.

### Prior adulticide use

At the time of this study (2021), the NWMAD had prior and ongoing use of truck-mounted ULV adulticide products Zenivex® E20 (active ingredient etofenprox; Central Life Sciences, Schaumburg, IL) and/or Anvil® 10 + 10 (active ingredient Sumithrin®; Clarke, St. Charles, IL). All sites received adulticide treatment once per year with either product prior to 2019. In 2019 and 2020, study sites AHN, DPS, and WHE received five treatments of Zenivex® E20 and Anvil® 10 + 10, respectively. No adulticide treatments were made in 2021. CDC bottle bioassays were completed in 2019 and 2020, where CFTs and topical applications were conducted in 2021.

### CDC bottle bioassays

Adult mosquitoes aged 3–7 days were evaluated for susceptibility to two pyrethroids (Sumithrin® and etofenprox) with the CDC bottle bioassay according to the CONUS Manual for Evaluating Insecticide Resistance in Mosquitoes Using the CDC Bottle Bioassay Kit guide [19]. CDC bottle bioassays were completed with either Sumithrin® or etofenprox due to prior use of these active ingredients. Therefore, deltamethrin was not included in CDC bottle bioassays. Technical grade insecticide (provided in the CDC kit) was diluted with acetone, according to the CONUS guide [19], to 20 ug/bottle for Sumithrin® and 12.5 ug/bottle for etofenprox. Insecticide solutions were stored at 4°C and were disposed of after one year. Bottles were treated with insecticide according to the CDC procedure and dried between four to six hours in a cool, dark place. Insecticide solutions were acclimated to room temperature before bottle treatment. Between 10–30 mosquitoes were aspirated into each 250 mL bottle and knockdown was recorded every 5 minutes for 45 minutes, then every 15 minutes until the completion of the bioassay at 120 minutes. The diagnostic times, the times at which >97% mortality should be observed in susceptible mosquitoes, for etofenprox and Sumithrin® were 15 and 30 minutes, respectively [19]. Reduced mortality at these times suggests IR [19].

### Caged field trials

Clarke-style bioassay cages [20] were constructed by cutting 6 inch (15.24 cm) diameter cardboard mailing tubes (Staples Inc., Framingham, MA) to 1.5 inch (3.81 cm) sections and drilling a 5/8 in (1.58 cm) hole in the side. Nylon tulle fabric (item number: 401703, Joann Stores LLC, Hudson, OH) was attached with hot glue to both faces. Between 10–30 mosquitoes were aspirated into the screened cage then plugged with a 5/8 inch (1.58 cm) steel end cap (Outwater Plastics Industries, Bogota, NJ). Mosquitoes were not anesthetized prior to aspiration. Cages of mosquitoes were attached 12 inch (30 cm) apart on rotating weathervanes mounted on a 1.5 m PVC pole [21].

Caged field trials were conducted in a large, open field at Maryville Academy in Des Plaines IL. Site preparation, mosquito processing, and data collection were performed in a similar manner to Britch et al. [22]. Prior to and during application, weather conditions were evaluated by a near-by weather station (WBAN:04838) and a hand-held anemometer (Ambient Weather WM-4 [Ambient Weather, Chandler, AZ]). For each spray, a spray line perpendicular to wind direction was identified and a 3 X 6 grid for cage deployment was set up down-wind. A total of 36 cages of mosquitoes were placed on weathervanes 15.2 m, 30.5 m, 45.7 m, 61.0 m, 76.2 m, and 91.4m (50 ft, 100 ft, 150 ft, 200 ft, 250 ft, and 300 ft, respectively) downwind from the path of the vehicle. Replicates were set in rows 15.2 m (50 ft) apart. One cage of field collected *Cx. pipiens* and one cage of lab susceptible *Cx. pipiens* were placed on each weathervane 20–30 minutes before adulticide application. A total of three sprays were performed.

Anvil® 10 + 10 was diluted 1:1 with mineral oil (5% Sumithrin® + 5% PBO) and sprayed in ultra-low volume (ULV) at 88.7 mL/min (3 oz/min) from a truck-mounted London Fog 18–20 (London Foggers, Minneapolis, MN) at 16 km/hr (10 mi/hr). Prior to application, droplet calibration was conducted with a DC-IV (KLD Labs, Hauppauge, NY). Each spray was performed at sunset when temperature inversion occurred [23]. Cages were retrieved after 10 minutes. Mosquitoes were aspirated to clean 473 ml (16 oz) paper cups (Solo Cup Company, Lake Forest IL) in the field to minimize excess adulticide exposure [24,25] and provided with a 10% sucrose solution. On arrival to the laboratory, mosquitoes were kept at ambient conditions on lab benches (22°C and 60% humidity) to avoid contamination of rearing facilities. Mortality and moribundity was recorded at 24 hours. Mortality was defined as lack of any movement when prompted with light blowing or tapping on the cage, and moribundity was defined as some body movement, an inability to fly, or inability to orient itself upright. Moribund mosquitoes were held for an additional 24 hours. All moribund mosquitoes had died by the second 24-hour interval, thus all moribund mosquitoes were analyzed as dead. Survivorship of non-moribund females during this 48-hour period remained unchanged.

### Topical application

Topical applications were conducted with technical grade insecticide according to a protocol by Norris et al. [26] that is also available on protocols.io [27]. Female mosquitoes at 3–8 days post-eclosion were aspirated into holding cages, anesthetized with carbon dioxide (CO_2_) gas for 30–60 seconds, then transferred to a glass petri dish on a chill table at -4 °C. Glass petri dishes were lined with filter paper that was replaced after each active ingredient. A 0.2 µL drop of insecticide solution was applied to the dorsal mesothorax using a 10 µL Hamilton® Gastight® syringe (Sigma-Aldrich, St. Louis, MO) fit with a 50-step repeating dispenser (Sigma-Aldrich, St. Louis, MO). The syringe was rinsed three times with acetone between active ingredients. Replicates were conducted in a low to high concentration gradient. Control mosquitoes were treated with acetone. After treatment, mosquitoes were placed in clean 473 ml (16 oz) paper cups and supplied with 10% sucrose solution and left in ambient conditions on lab benches (22°C and 60% humidity). Mortality and moribundity, as described above, were recorded after 24 hours. Moribund mosquitoes were held for an additional 24 hours to ensure mortality and were analyzed as dead. At least five different concentrations of each insecticide were applied to a minimum of three replicates of each population. Mosquito populations that did not reach this threshold due to insufficient egg collection were omitted from analysis (AHS).

Three active ingredients, representing each class of pyrethroid, were selected for testing: Sumithrin® (d-phenothrin) (Class I pyrethroid), deltamethrin (Class II pyrethroid), and etofenprox (non-ester pyrethroid) (Sigma-Aldrich, St. Louis, MO). Insecticides were diluted with acetone to a range of concentrations that encompass the necessary concentrations to fit a dose response curve. Unsynergized insecticides were diluted to 0.01–10,000 ppm (0.002–2000 ng/droplet). Synergized insecticide solutions were created with sublethal doses of piperonyl butoxide (PBO); this synergist is very common in formulated adulticide products, including the adulticide used for CFTs (Anvil® 10 + 10). Sublethal doses of PBO resulted in <5% mortality. The PBO dose for field collected specimens was 75 ppm (15 ng/droplet) and 25 ppm (5 ng/droplet) for lab susceptible mosquitoes. Insecticides for the synergized solutions were diluted to 0.0001–5000 ppm (0.00002–1000 ng/droplet). All insecticide solutions were stored at 4°C and acclimated to room temperature before use.

### Analysis

Mortality corrections were performed for all assays using Abbott’s formula [28]. All field sites were combined for analysis, but site-specific results are available separately in the supporting information. For CDC bottle bioassays, Kaplan-Meier survival curves and mean mortality (proportion dead) at diagnostic time and the end of the assay were calculated and are reported. For caged field trials, mean mortality (proportion dead) at each distance from the spray line are reported and significant differences between field-collected and lab susceptible cages were detected with a two-tailed t-test. A probit analysis [29–31] was used to calculate the LC_50_ for each active ingredient and each population. Resistance ratios (RR) were calculated as LC_50_ of field-collected mosquitoes divided by LC_50_ of lab susceptible mosquitoes and categorized for resistance intensity [32]. All analyses were calculated using R Studio [33] with packages “survival” [34] and “ecotox” [35] and visualized with “ggplot2” [36].

## Results

### CDC bottle bioassays

**Fig 2:**
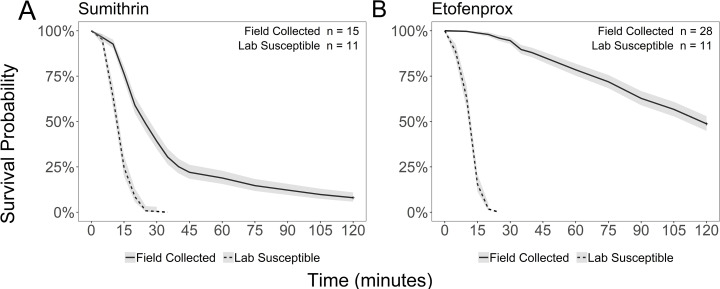
CDC bottle bioassay Kaplan-Meier survival probability curves. 95% confidence intervals for field-collected and lab susceptible *Cx. pipiens*. Active ingredients used were etofenprox (A) and Sumithrin® (B). Diagnostic time for etofenprox is 15 minutes (12.5 ug/bottle) and 30 minutes for Sumithrin® (20 ug/bottle).

Field populations of *Cx. pipiens* did not reach >90% mortality by the diagnostic time for etofenprox nor Sumithrin®, indicating that IR may be present based on the CONUS guide. Overall, 11% mortality was achieved at the diagnostic time for etofenprox, and 61% mortality at the diagnostic time for Sumithrin® (Fig 2, Table 1). For etofenprox, complete mortality in field collected populations was never reached by the end of the two-hour bioassay (Table 1). In contrast, the susceptible strain of *Cx. pipiens* reached complete mortality by the diagnostic time for Sumithrin® (Fig 2, Table 1). In lab susceptible mosquitoes, only 90% mortality was observed at the diagnostic time for etofenprox (Fig 2, Table 1). Since this *Cx. pipiens* strain has been in isolation for decades, it is extremely unlikely that IR is present. Rather, the diagnostic time of 15 minutes may not be long enough. Surprisingly, there were differences in mortality at the diagnostic time and at the end of the assay between populations from different sites, despite these sites being geographically close to one another (S1 Fig).

**Table 1:** Mortality in lab susceptible and field collected *Cx. pipiens* according to CDC bottle bioassay at diagnostic times (DT) and end of assay.

Population	Etofenprox			Sumithrin®		
	n	Mortality at 15 min (DT) ± SE	Mortality at 120 min ± SE	n	Mortality at 30 min (DT) ± SE	Mortality at 120 min ± SE
Lab susceptible	11	0.90 ± 0.03	1.00 ± 0.00	11	0.99 ± 0.01	1.00 ± 0.00
Field collected	28	0.11 ± 0.05	0.50 ± 0.05	21	0.61 ± 0.05	0.91 ± 0.03

Mortality values are the proportion dead with standard error (±SE). n is the number of CDC bottle bioassay replicates.

### Caged field trials

**Fig 3:**
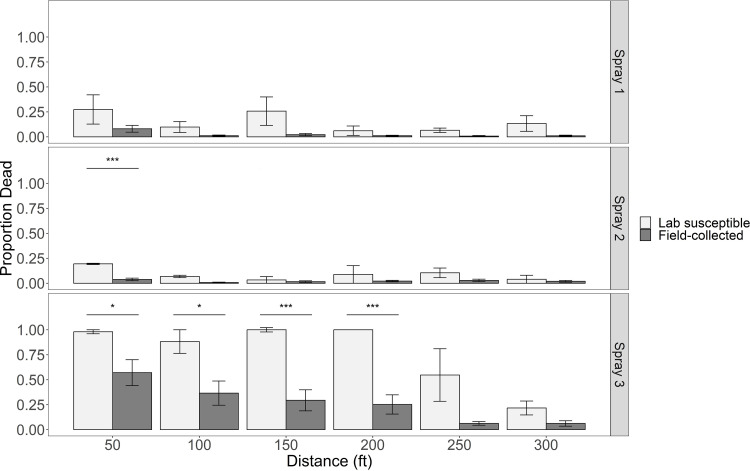
Mean mortality of field-collected and lab susceptible colony *Cx. pipiens* from caged field trials. Error bars indicate standard error. Significant differences detected with two-tailed t-tests (* p < 0.05, *** p < 0.001).

Despite meeting minimum weather conditions, sprays 1 and 2 resulted in low mortality in both lab susceptible and field-collected mosquitoes (Fig 3). Droplet calibration resulted in a volume median diameter of 13.8 microns. In spray 3, > 95% mortality was observed for lab susceptible mosquitoes at 15.2 m, 30.5 m, 45.7 m, and 61.0 m (50 ft, 100 ft, 150 ft, and 200 ft) from the spray line (Fig 3) whereas 47% (± 13.0%), 32% (± 11.8%), 35% (± 13.9%), and 21% (± 8.2%) mortality was observed in field-collected mosquitoes at the same distances, respectively. Mortality in field-collected populations of *Cx. pipiens* was significantly lower than lab susceptible mosquitoes at three of these distances (Fig 3). In using the same mortality cutoff values as the CDC bottle bioassay, field-collected mosquitoes did not reach >90% mortality at the distances where >95% mortality was observed in lab susceptible mosquitoes, indicating IR. Truck-mounted ULV adulticides should drift to 300 ft but low mortality at 250 and 300 ft in the lab susceptible mosquitoes during spray 3 provides evidence that the amount of product reaching these distances is insufficient (Fig 3). There were differences in mortality between populations from different sites at the same distances in each spray (S1 Table).

### Topical application

Field-collected *Cx. pipiens* demonstrated resistance to all six insecticide solutions tested when compared to a susceptible lab strain. RRs ranged from 7.2–119.9 (low to high resistance) for unsynergized active ingredients, and 9.9–22.5 (low to moderate resistance) for synergized active ingredients. LC_50_ values for all active ingredients were lower when PBO was added, contrary to some increases in the RR (Table 2).

**Table 2:** Toxicity of six tested insecticides to field collected *Cx. pipiens* compared to lab susceptible mosquitoes by topical application.

Active Ingredient	n	Slope ± SE	LC_50_ (95%CI)	χ^2^	RR^b^	RC^c^
Unsynergized Etofenprox	1285	1.16 ± 0.06	8.56 (1.17 - 22.76)	1481.64	7.8	Low
^a^Synergized Etofenprox	1181	0.93 ± 0.049	3.27 (1.98 - 5.53)	290.82	9.9	Low
Unsynergized Sumithrin®	1421	1.16 ± 0.06	8.89 (3.31 - 17.79)	1041.56	7.2	Low
^a^Synergized Sumithrin®	1165	1.23 ± 0.069	7.83 (5.13 - 12.28)	278.22	19.6	Moderate
Unsynergized Deltamethrin	953	1.66 ± 0.09	0.56 (0.38 - 0.77)	150.61	119.9	High
^a^Synergized Deltamethrin	930	0.98 ± 0.06	0.07 (0.04 - 0.16)	278.16	22.5	Moderate

LC_50_ in ng active ingredient/mg mosquito (average weight is 3.18 mg/female). Probit analysis output includes the number of females tested, slope and standard error, LC_50_ value and 95% confidence intervals, and χ^2^ value.

^a^Synergized active ingredients contained a 15 ng dose of PBO.

^b^Resistance ratio = LC_50_ of field-collected mosquitoes/LC50 of susceptible lab strain.

^c^Resistance classification (Kim et al. 2004): RR < 10 = low, 10–40 = moderate, 40–160 = high, > 160 = extremely high.

Mosquitoes exhibited highest susceptibility to synergized deltamethrin (0.07 ng/mg), followed closely by unsynergized deltamethrin (0.56 ng/mg) (Table 2). These solutions elicited the highest RRs of 22.5 and 119.9 respectively. High RRs to both unsynergized and synergized deltamethrin were unexpected, as this active ingredient has never been used by the NWMAD, though the extent of private company and homeowner use of deltamethrin is unknown. Since this information is unknown, cross-resistance amongst the pyrethroid family is likely at play in these field-collected populations. Synergized etofenprox (3.27 ng/mg, 9.9 RR), synergized Sumithrin® (7.83 ng/mg, 19.6 RR), unsynergized etofenprox (8.56 ng/mg, 7.8 RR), and unsynergized Sumithrin® (8.89 ng/mg, 7.2 RR) followed distantly behind in toxicity and comparative resistance (Table 2).

Each site roughly followed the same patterns in active ingredient toxicity when analyzed separately, but there is a wide range of LC_50_ values amongst populations (S2 Table). When separated by site, synergized and unsynergized deltamethrin remained the most potent insecticide solutions, with LC_50_ values ranging from 0.02–0.07 ng/mg and 0.31–1.02 ng/mg, respectively (S2 Table). As expected, synergized Sumithrin® and synergized etofenprox both resulted in a lower LC_50_ concentration than their unsynergized counterparts (4.79–19.25 ng/mg and 1.94–4.87 ng/mg, respectively) (S2 Table).

## Discussion

**Fig 4:**
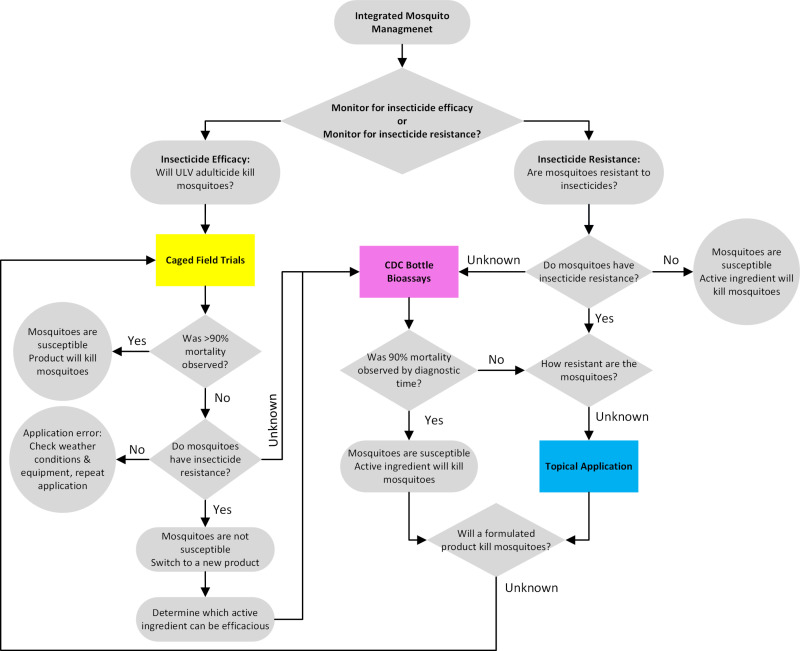
Categorical decision-making tree for assay selection for evaluating insecticide efficacy and resistance. Rounded squares indicate root nodes (questions and supporting information), squares indicate processes (assays), diamonds indicate decision nodes (yes/no/unknown) that branch to root and terminal nodes, and circles indicate terminal nodes (outcomes and conclusions).

Increasing incidence of IR in mosquito vector populations presents a significant threat to successful operational mosquito control and thereby prevention of diseases like WNV. We used three phenotypic assays to assess IR in *Cx. pipiens* populations from six neighborhoods in the NWMAD, located in Cook County, IL, USA. Our results from CDC bottle bioassays revealed that IR may be present in field-collected populations, but further examination was needed to understand the extent of IR. Using CFTs, we assessed the impact of formulated insecticides broadcast by truck-mounted ULV sprays on field-collected populations, and likewise observed IR as compared to a susceptible laboratory colony at distances with adequate treatment. We then conducted direct toxicological testing of active ingredients using topical assays; the resulting resistance ratios calculated facilitated a quantitative understanding of resistance in each study site. Overall, with the culmination of three assays applied to field-collected mosquitoes, we conclude that applications of *any* pyrethroid-based adulticide will provide subpar adult control for *Cx. pipiens* in the Chicago area due to IR. Given the many approaches used for IR assessment and based on our comprehensive approach, we provide a decision-making tool (Fig 4), complete with a list of advantages and disadvantages of each assay (Table 3), to assist MCOs in choosing the most appropriate and informative IR assay. Because evaluating insecticide efficacy and resistance require different assays, we conclude that a holistic understanding of IR depends on using a multi-step approach, starting with CDC bottle bioassays, and continuing with topical applications and CFTs if IR is detected.

**Table 3:** Advantages and disadvantages of caged field trials, CDC bottle bioassays, and topical applications for insecticide efficacy and resistance testing.

Caged Field Trials	CDC Bottle Bioassay	Topical Applications
Pros: Determine if formulated adulticide product will kill mosquitoes Suggests the presence of insecticide resistance Not limited by mosquito species or age Track efficacy changes in population over time	Pros: Suggests the presence of insecticide resistance Easily test many active ingredients Easily accessible and portable (free kits available from CDC) Track resistance changes in population over time	Pros: Identify and quantify the level of insecticide resistance Not limited by mosquito species or age Easily test many active ingredients and concentrations Easily accessible and portable (low cost) Track resistance changes in population over time
Cons: Dependent on abiotic conditions (temperature, wind speed & direction, etc.) Can only test formulated adulticide products Difficult to switch between products and concentrations Time, equipment, and personnel intensive Results are not directly related to impacts on field populations Method of exposure not relevant to barrier treatments Cannot quantify insecticide resistance	Cons: Limited by mosquito species and age Need a reference population if species does not have a diagnostic time Cannot use formulated adulticide products Limited to one concentration of active ingredient Cannot quantify insecticide resistance Method of exposure not relative to ULV adulticide Cannot determine if formulated ULV adulticide with same active ingredient will kill mosquitoes	Cons: Need a reference population for comparison Need experience with serial dilutions and concentration calculations Many replicates needed Method of exposure not relevant to barrier treatments Cannot determine if formulated ULV adulticide with same active will kill mosquitoes

By design, the CDC bottle bioassay is the most restrictive assay for IR used in this study (Fig 4). In using standardized technical grade active ingredients and concentrations, this assay facilitates assessment of IR in mosquito strains across geographic regions (Table 3). The CDC bottle bioassay method of exposure, prolonged tarsal contact, more closely approximates barrier treatments than ULV adulticide applications [37]. In addition, the dosage of insecticide is highly variable due to differences in time spent flying compared to standing, so exposure among individuals varies and more replicates are necessary [38,39]. Similar criticisms have been made of the WHO susceptibility tube assays [40]. Established diagnostic times in the CDC bottle bioassay manual for the CONUS are limited to five vector species (*Aedes aegypti* L., *Ae. albopictus* Skuse, *Cx. pipiens*, *Cx. quinquefasciatus* Say, and *Cx. tarsalis* Coquillett) and do not include other vector species or nuisance species that are often the focus of mosquito control efforts [19]. In the case of species without a pre-established diagnostic time, it is recommended to use the times of a closely related species, though diagnostic times between species in the same genus can vary considerably [19], making this decision impractical. Furthermore, diagnostic times defined for particular species may not be informative for field-collected specimens with complex genetics resulting from hybridization, which is not supported by the CDC bottle bioassay. In this study, CDC bottle bioassays were completed under the assumption that species hybridization is not present in field-collected populations, however this is well-documented amongst the *Cx. pipiens* complex in the Chicago area [41,42].

Operationally, CFTs answer the fundamental question of product effectiveness in field-collected mosquitoes under ideal field conditions, but have limitations in IR monitoring (Table 3). It is imperative to recognize that observations of complete mortality in CFTs may not directly correlate to successful knockdown of field-collected populations of mosquitoes in nature. However, it is interesting that the mean proportion dead of field-collected mosquitoes at 50 ft (0.57, Fig 3) is similar to the reduction in host-seeking mosquitoes after adulticide applications (IRR of 0.46) from the same sites in 2019 and 2020 [10], though more work is needed to fully characterize this relationship. CFTs are the most costly of the assays utilized in this study, requiring purchase of the adulticide application equipment, training and licensure of personnel, and the adulticide product of choice which is often only available in large quantities. Even within a well-established MCO, preparation of weathervanes/stands, mosquito cages, mosquito rearing, finding a suitable application location, and setting up the grid is very time and resource intensive. The method of exposure is complex and highly susceptible to environmental conditions, as was evident in sprays 1 and 2 (Fig 3). Therefore, adulticide droplet metrics or direct comparisons to a susceptible strain of mosquitoes are needed to differentiate between lack of exposure from presence of IR when reduced mortality is observed (Fig 3). However, it is often impossible for a mosquito control district to find a susceptible field-collected population to compare to [43] and droplet analysis methods can be costly in time and/or finances. Use of other assays like the CDC bottle bioassay or topical applications, in lieu of a lab-susceptible population for CFTs, can inform the mechanism behind reduced mortality observations.

Topical application is the most flexible assay tested for choice of active ingredient, concentration, and mosquito species, but does require a lab-susceptible population for calculating RRs. Operationally, this assay can be used as a high-throughput method to quantify susceptibility to many active ingredients, allowing for consideration of both active ingredient and concentration in future product purchase (Fig 4). While materials for topical applications are not free like the CDC bottle bioassay kit, they are similarly favorable in cost and portability (Table 3) [38]. Although this assay does not address the issues surrounding species hybridization, the outcome from topical applications is exact dose of insecticide needed to kill field-collected mosquitoes, regardless of their genetics or comparison to susceptible populations. Topical application was the most informative assay for IR monitoring, as our results revealed IR across populations of *Cx. pipiens* exposed to all three pyrethroids, even when synergized with PBO (Table 2). Maintenance of resistance after the addition of a synergist indicates that resistance may be due to a target site mutation, as oxidase activity is inhibited by PBO, though other mechanisms are possible. The perpetuation of resistance with the addition of PBO may have profound ramifications for vector control, as the recommendation of adding a synergist to adulticide products for targeting resistant mosquitoes may be insufficient, resulting in poor control. Our results were paralleled by a study reporting low to moderate levels of resistance to permethrin in field-collected *Cx. pipiens* in the United States [44]. It remains unclear how quickly levels of resistance can change in field-collected *Cx. pipiens* populations. Extremely high levels of resistance to many pyrethroids were documented in *Cx. pipiens pallens* from South Korea and Japan [32,45]. Our results reveal that while field-collected *Cx. pipiens* populations mirrored one another across sites in toxicant dosage response (S2 Table), variation provides new insights into IR across a fine geographic scale. Data generated by our topical application assay highlights the importance of appreciating the IR potential of *Cx. pipiens* mosquitoes and its implications for vector control.

The widespread use of pyrethroids for pest control in agricultural and residential settings can contribute to selection pressure for resistance [46]. As more reports of IR in vector populations are published each year, the reality of finding a product that will be effective in killing field-collected mosquitoes is dwindling based on the number of products that are registered for use by the EPA. Adding to this challenge, there are only two chemical classes with different modes of action registered for mosquito control use: pyrethroids and organophosphates. Therefore, when IR is detected, the options available to MCOs are few, and the *only* recommendation after detecting IR is to switch to a product with a new mode of action [2]. This points to the necessity of IR monitoring to find a product that will work, and to keep that effective product available for disease control emergencies. Based on the results from three different IR assays, it is clear that field-collected populations of *Cx. pipiens* the NWMAD are resistant, or at least tolerant, to multiple pyrethroids. Thus, these active ingredients will not be as efficacious as intended during adulticide application.

The experience of implementing multiple phenotypic assays, scrutinizing the strengths and weaknesses of each assay, and translating the data to adulticide effectiveness in the field (Table 3), informed the guide to decision-making for IR assay implementation presented here (Fig 4). Choosing which assay(s) to implement in insecticide efficacy and resistance testing is largely determined by personnel, equipment, and the goals and information being inferred from experimentation. This simplified but straightforward guide allows the user to make quick and informed choices about IR assay selection.

## Supporting information

S1 Fig CDC bottle bioassay Kaplan-Meier survival probability curves with 95% confidence intervals for each field-collected *Cx. pipiens* population. Active ingredients used were etofenprox (A) and Sumithrin® (B). Diagnostic time for etofenprox is 15 minutes and 30 minutes for Sumithrin®.
(JPEG)

S1 Table Mortality of field-collected Cx. pipiens from caged field trials. Data displayed according to spray event (spray number) and distance from the spray line. n is the number of female mosquitoes in each cage.
(PDF)

S2 Table Toxicity of six insecticides to lab susceptible strain and five field-collected strains by topical application. Synergized active ingredients contained a 15 ng dose of PBO for sites AHN, AHC, DPS, DPN, and WHE (field collected populations). Synergized active ingredients contained a 5 ng dose of PBO for LAB (lab susceptible strain). LC_50_ in ng active ingredient/mg mosquito was calculated via probit analysis (average weight is 3.18 mg/female for field collected mosquitoes and 2.65 mg/female for lab susceptible mosquitoes). Analysis output includes number of mosquitoes tested (n), slope and standard error, LC_50_ value and 95% confidence interval, and χ^2^ value. Resistance ratios (RR) were calculated by comparing LC_50_ values between the field-collected site (AHN, AHC, DPS, DPN, or WHE) with the lab susceptible strain (LAB).
(PDF)

## References

[1] Beard CB, Visser SN, Petersen LR. The Need for a National Strategy to Address Vector-Borne Disease Threats in the United States. J Med Entomol. 2019;56(5):1199–203. doi: 10.1093/jme/tjz074 31505668 PMC7058377

[2] [AMCA] American Mosquito Control Association. Best Practices for Integrated Mosquito Management. 2021.

[3] Burtis JC, Poggi JD, McMillan JR, Crans SC, Campbell SR, Isenberg A, et al. NEVBD Pesticide Resistance Monitoring Network: Establishing a Centralized Network to Increase Regional Capacity for Pesticide Resistance Detection and Monitoring. J Med Entomol. 2021;58(2):787–97. doi: 10.1093/jme/tjaa236 33128057 PMC13032001

[4] Dubie TR, Bartholomay L, Clifton M, Walker ED. Variation in Susceptibility to Permethrin in Culex pipiens and Culex restuans Populations in the Great Lakes Region of the United States. J Am Mosq Control Assoc. 2022;38(3):188–97. doi: 10.2987/22-7062 35901310

[5] Bertolotti L, Kitron UD, Walker ED, Ruiz MO, Brawn JD, Loss SR, et al. Fine-scale genetic variation and evolution of West Nile Virus in a transmission “hot spot” in suburban Chicago, USA. Virology. 2008;374(2):381–9. doi: 10.1016/j.virol.2007.12.040 18261758

[6] Mutebi J-P, Delorey MJ, Jones RC, Plate DK, Gerber SI, Gibbs KP, et al. The impact of adulticide applications on mosquito density in Chicago, 2005. J Am Mosq Control Assoc. 2011;27(1):69–76. doi: 10.2987/10-6045.1 21476450

[7] Clifton ME, Xamplas CP, Nasci RS, Harbison J. Gravid Culex pipiens Exhibit A Reduced Susceptibility to Ultra-Low Volume Adult Control Treatments Under Field Conditions. J Am Mosq Control Assoc. 2019;35(4):267–78. doi: 10.2987/19-6848.1 31922942

[8] Sass D, Li B, Clifton M, Harbison J, Xamplas C, Smith R. The Impact of Adulticide on Culex Abundance and Infection Rate in North Shore of Cook County, Illinois. J Am Mosq Control Assoc. 2022;38(1):46–58. doi: 10.2987/21-7036 35276731

[9] Lopez K, Irwin P, Bron GM, Paskewitz S, Bartholomay L. Ultra-low volume (ULV) adulticide treatment impacts age structure of Culex species (Diptera: Culicidae) in a West Nile virus hotspot. J Med Entomol. 2023;60(5):1108–16. doi: 10.1093/jme/tjad088 37473814

[10] Lopez K, Susong K, Irwin P, Paskewitz S, Bartholomay L. Impacts of ground ultra-low volume adulticide applications on Culex pipiens and Culex restuans (Diptera: Culicidae) abundance, age structure, and West Nile virus infection in Cook County, Illinois. J Med Entomol. 2024;61(4):1043–53. doi: 10.1093/jme/tjae041 38527268 PMC12102602

[11] Burgess ER 4th, Lopez K, Irwin P, Jaeger CP, Estep AS. Assessing pyrethroid resistance status in the Culex pipiens complex (Diptera: Culicidae) from the northwest suburbs of Chicago, Illinois using Cox regression of bottle bioassays and other detection tools. PLoS One. 2022;17(6):e0268205. doi: 10.1371/journal.pone.0268205 35767519 PMC9242439

[12] Brogdon WG, McAllister JC. Insecticide resistance and vector control. Emerg Infect Dis. 1998;4(4):605–13. doi: 10.3201/eid0404.980410 9866736 PMC2640263

[13] Lofgren CS, Anthony DW, Mount GA. Size of aerosol droplets impinging on mosquitoes as determined with a scanning electron microscope. J Econ Entomol. 1973;66(5):1085–8. doi: 10.1093/jee/66.5.1085 4747954

[14] Cooperband MF, Golden FV, Clark GG, Jany W, Allan SA. Prallethrin-induced excitation increases contact between sprayed ultralow volume droplets and flying mosquitoes (Diptera: Culicidae) in a wind tunnel. J Med Entomol. 2010;47(6):1099–106. doi: 10.1603/me10021 21175059

[15] Zhang H, Dorr GJ, Hewitt AJ. Retention and efficacy of ultra-low volume pesticide applications on Culex quinquefasciatus (Diptera: Culicidae). Environ Sci Pollut Res Int. 2015;22(21):16492–501. doi: 10.1007/s11356-015-5480-9 26423287

[16] Aldridge RL, Kaufman PE, Bloomquist JR, Gezan SA, Linthicum KJ. Impact of Topical Application Site On the Efficacy of Permethrin and Malathion To Culex quinquefasciatus. J Am Mosq Control Assoc. 2016;32(4):300–7. doi: 10.2987/16-6584.1 28206864

[17] Aldridge RL, Kaufman PE, Bloomquist JR, Gezan SA, Linthicum KJ. Application Site and Mosquito Age Influences Malathion- and Permethrin-Induced Mortality in Culex quinquefasciatus (Diptera: Culicidae). J Med Entomol. 2017;54(6):1692–8. doi: 10.1093/jme/tjx160 28968685

[18] Darsie RF, Ward RA. Identification and geographical distribution of the mosquitoes of North America, north of Mexico. Fresno, California: American Mosquito Control Association. 1981.

[19] Centers for Disease Control and Prevention C. Conus guideline for evaluating insecticide resistance in vectors using the CDC bottle bioassay. 2020.

[20] Fritz BK, Hoffmann WC, Farooq M, Walker T, Bonds J. Filtration effects due to bioassay cage design and screen type. J Am Mosq Control Assoc. 2010;26(4):411–21. doi: 10.2987/10-6031.1 21290937

[21] Clayson PJ, Latham M, Bonds JAS, Healy SP, Crans SC, Farajollahi A. A droplet collection device and support system for ultra-low-volume adulticide trials. J Am Mosq Control Assoc. 2010;26(2):229–32. doi: 10.2987/10-5999.1 20649136

[22] Britch SC, Linthicum KJ, Aldridge RL, Golden FV, Walker TW. Visualizing Efficacy of Pesticides Against Disease Vector Mosquitoes in the Field. J Vis Exp. 2019;(145):10.3791/58440. doi: 10.3791/58440 30933054

[23] Bonds JAS. Ultra-low-volume space sprays in mosquito control: a critical review. Med Vet Entomol. 2012;26(2):121–30. doi: 10.1111/j.1365-2915.2011.00992.x 22235908

[24] Bunner BL, Perich MJ, Boobar LR. Culicidae (Diptera) mortality resulting from insecticide aerosols compared with mortality from droplets on sentinel cages. J Med Entomol. 1989;26(3):222–5. doi: 10.1093/jmedent/26.3.222 2566687

[25] Barber JAS, Greer M, Coughlin J. The Effect of Pesticide Residue on Caged Mosquito Bioassays. Journal of the American Mosquito Control Association. 2006;22(3):469–72. doi: 10.2987/8756-971x(2006)22[469:teopro]2.0.co;2 17067048

[26] Norris EJ, Gross AD, Dunphy BM, Bessette S, Bartholomay L, Coats JR. Comparison of the Insecticidal Characteristics of Commercially Available Plant Essential Oils Against Aedes aegypti and Anopheles gambiae (Diptera: Culicidae). J Med Entomol. 2015;52(5):993–1002. doi: 10.1093/jme/tjv090 26336230

[27] J. Norris E, R. Coats J. Topical Application of Insecticidal Active Ingredients v1. Springer Science and Business Media LLC. 2018. doi: 10.17504/protocols.io.q7idzke

[28] Abbott WS. A method of computing the effectiveness of an insecticide. J Econ Entomol. 1925;18(2):265–7.

[29] Finney DJ. Probit analysis. Cambridge, England: Cambridge University Press. 1971.

[30] Wheeler MW, Park RM, Bailer AJ. Comparing median lethal concentration values using confidence interval overlap or ratio tests. Environ Toxicol Chem. 2006;25(5):1441–4. doi: 10.1897/05-320r.1 16704080

[31] Robertson JL, Savin NE, Russell RM, Preisler HK. Bioassays with arthropods. Boca Raton (FL): CRC press; 2007. ISBN: 9780849323317.

[32] Kim N-J, Chang K-S, Lee W-J, Ahn Y-J. Monitoring of Insecticide Resistance in Field-Collected Populations of Culex pipiens pallens (Diptera: Culicidae). Journal of Asia-Pacific Entomology. 2007;10(3):257–61. doi: 10.1016/s1226-8615(08)60360-x

[33] R Development Core Team, Posit PBC. Version 4.2.0. 2023. https://r-studio.com

[34] Therneau TM, Lumley T, Atkinson E, Crowson C. Survival: Survival Analysis. 2023. Available from: https://CRAN.R-project.org/package=survival

[35] Hlina BL. Ecotox: Analysis of Ecotoxicology. 2021. Available from: https://CRAN.R-project.org/package=ecotox

[36] Wickham H, Chang W, Henry L, Pedersen T, Takahashi K, Wilke C. Create elegant data visualization using the grammar of graphics. 2023. Available from: https://ggplot2.tidyverse.org

[37] Richards SL, Byrd BD, Reiskind MH, White AV. Assessing Insecticide Resistance in Adult Mosquitoes: Perspectives on Current Methods. Environ Health Insights. 2020;14:1178630220952790. doi: 10.1177/1178630220952790 32952401 PMC7477762

[38] Althoff RA, Huijben S. Comparison of the variability in mortality data generated by CDC bottle bioassay, WHO tube test, and topical application bioassay using Aedes aegypti mosquitoes. Parasit Vectors. 2022;15(1):476. doi: 10.1186/s13071-022-05583-2 36539831 PMC9769033

[39] Waits CM, Fulcher A, Louton JE, Richardson AG, Becnel JJ, Xue R, et al. A Comparative Analysis of Resistance Testing Methods inAedes albopictus(Diptera: Culicidae) from St. Johns County, Florida. Florida Entomologist. 2017;100(3):571–7. doi: 10.1653/024.100.0313

[40] Bagi J, Grisales N, Corkill R, Morgan JC, N’Falé S, Brogdon WG, et al. When a discriminating dose assay is not enough: measuring the intensity of insecticide resistance in malaria vectors. Malar J. 2015;14:210. doi: 10.1186/s12936-015-0721-4 25985896 PMC4455279

[41] Aardema ML, vonHoldt BM, Fritz ML, Davis SR. Global evaluation of taxonomic relationships and admixture within the Culex pipiens complex of mosquitoes. Parasit Vectors. 2020;13(1):8. doi: 10.1186/s13071-020-3879-8 31915057 PMC6950815

[42] Kothera L, Mutebi J-P, Kenney JL, Saxton-Shaw K, Ward MP, Savage HM. Bloodmeal, Host Selection, and Genetic Admixture Analyses of Culex pipiens Complex (Diptera: Culicidae) Mosquitoes in Chicago, IL. J Med Entomol. 2020;57(1):78–87. doi: 10.1093/jme/tjz158 31576405 PMC11313203

[43] Richards SL, Byrd BD, Breidenbaugh M, Vandock K. Survey of United States Mosquito Control Programs Reveals Opportunities to Improve the Operational Value of Centers for Disease Control and Prevention Bottle Bioassays. J Med Entomol. 2022;59(5):1827–30. doi: 10.1093/jme/tjac076 35751624

[44] Bearden SL. Evaluation of insecticide resistance in Louisiana adult mosquitoes and the efficacies of selected larvicides. Baton Rouge (LA): Louisiana State University. 2000.

[45] Yang YC, Kim H, Jang C, Chang K. Insecticide susceptibility of Culex pipiens pallens (L.) and Aedes albopictus (Skuke) to five commonly used pesticides in the Republic of Korea. Entomological Research. 2019;49(11):477–82. doi: 10.1111/1748-5967.12339

[46] Scott JG, Yoshimizu MH, Kasai S. Pyrethroid resistance in Culex pipiens mosquitoes. Pestic Biochem Physiol. 2015;120:68–76. doi: 10.1016/j.pestbp.2014.12.018 25987223

