# Supplementary material for: A multi-assay assessment of insecticide resistance in Culex pipiens (Diptera: Culicidae) informs a decision-making framework
Source: PLoS One. 2025 Jun 9;20(6):e0324194. doi: 10.1371/journal.pone.0324194 (PMC12148167; doi:10.1371/journal.pone.0324194)
Supplement: S1 Table — Data displayed according to spray event (spray number) and distance from the spray line. n is the number of female mosquitoes in each cage. (PDF) [file pone.0324194.s002.pdf]

S1 Table. Mortality of field collected *Cx. pipiens* from caged field trials. Data displayed according to spray event (spray number) and distance from the spray line. n is the number of female mosquitoes in each cage.

Spray Number	Site	Proportion Dead at 50 ft (n)	Proportion Dead at 100 ft (n)	Proportion Dead at 150 ft (n)	Proportion Dead at 200 ft (n)	Proportion Dead at 250 ft (n)	Proportion Dead at 300 ft (n)
1	AHC	0.14 (7)	0.00 (9)	0.00 (12)	0.00 (9)	0.00 (12)	0.07 (14)
	AHN	0.04 (24)	0.00 (22)	0.00 (22)	0.00 (22)	0.00 (21)	0.00 (20)
	AHS	0.00 (17)	0.00 (12)	0.00 (12)	0.00 (16)	0.00 (19)	0.00 (15)
	DPN	0.08 (25)	0.00 (29)	0.00 (8)	0.03 (26)	0.04 (21)	0.00 (28)
	DPS	0.00 (7)	0.00 (5)	-	0.00 (10)	0.00 (12)	0.00 (16)
	WHE	0.22 (18)	0.00 (14)	0.00 (8)	0.04 (21)	0.00 (18)	0.00 (28)
2	AHC	0.00 (30)	0.00 (28)	0.00 (31)	0.00 (30)	0.07 (28)	0.03 (31)
	AHN	0.00 (25)	0.00 (23)	0.00 (20)	0.04 (22)	0.00 (21)	0.00 (21)
	AHS	0.03 (33)	0.03 (33)	0.06 (31)	0.00 (32)	0.06 (30)	0.03 (33)
	DPN	0.00 (34)	0.00 (26)	0.00 (30)	0.00 (31)	0.00 (33)	0.00 (26)
	DPS	0.08 (34)	0.00 (35)	0.00 (29)	0.02 (35)	0.00 (32)	0.00 (27)
	WHE	0.07 (26)	0.00 (26)	0.00 (25)	0.03 (26)	0.04 (25)	0.03 (31)
3	AHC	0.25 (28)	0.58 (31)	0.59 (27)	0.16 (25)	0.00 (24)	0.12 (24)
	AHN	0.35 (20)	0.29 (24)	0.04 (35)	0.08 (24)	-	0.00 (19)
	AHS	0.40 (25)	0.03 (26)	0.03 (26)	0.08 (24)	0.00 (19)	0.00 (19)
	DPN	1.00 (24)	0.76 (26)	0.78 (32)	0.50 (20)	0.05 (20)	0.05 (18)
	DPS	0.68 (29)	0.11 (27)	0.12 (25)	0.40 (22)	0.03 (30)	0.00 (24)
	WHE	0.13 (30)	0.15 (33)	0.82 (29)	0.00 (30)	0.04 (21)	0.10 (30)
