# Supplementary material for: A multi-assay assessment of insecticide resistance in Culex pipiens (Diptera: Culicidae) informs a decision-making framework
Source: PLoS One. 2025 Jun 9;20(6):e0324194. doi: 10.1371/journal.pone.0324194 (PMC12148167; doi:10.1371/journal.pone.0324194)
Supplement: S2 Table — Synergized active ingredients contained a 15 ng dose of PBO for sites AHN, AHC, DPS, DPN, and WHE (field collected populations). Synergized active ingredients contained a 5 ng dose of PBO for LAB (lab susceptible strain). LC50 in ng active ingredient/mg mosquito was calculated via probit analysis (average weight is 3.18 mg/female for field collected mosquitoes and 2.65 mg/female for lab susceptible mosquitoes). Analysis output includes number of mosquitoes tested (n), slope and standard error, LC50 value and 95% confidence interval, and χ2 value. Resistance ratios (RR) were calculated by comparing LC50 values between the field-collected site (AHN, AHC, DPS, DPN, or WHE) with the lab susceptible strain (LAB). (PDF) [file pone.0324194.s003.pdf]

S2 Table. Toxicity of six insecticides to lab susceptible strain and five field collected strains by topical application. Synergized active ingredients contained a 15 ng dose of PBO for sites AHN, AHC, DPS, DPN, and WHE (field collected populations). Synergized active ingredients contained a 5 ng dose of PBO for LAB (lab susceptible strain). LC_{50} in ng active ingredient/mg mosquito was calculated via probit analysis (average weight is 3.18 mg/female for field collected mosquitoes and 2.65mg/female for lab susceptible mosquitoes). Analysis output includes number of mosquitoes tested (n), slope and standard error, LC_{50} value and 95% confidence interval, and χ^2 value. Resistance ratios (RR) were calculated by comparing LC_{50} values between the field-collected site (AHN, AHC, DPS, DPN, or WHE) with the lab susceptible strain (LAB).

Active Ingredient	Site	n	Slope ± SE	LC_{50} (95% CI)	χ^2	RR
Unsynergized Etofenprox	LAB	796	0.87 ± 0.09	1.09 (0.022 - 14.406)	1111.15	1.0
	AHN	255	1.29 ± 0.16	8.11 (4.635 - 12.415)	5.42	7.4
	AHC	191	2.75 ± 0.430	26.07 (19.154 - 34.629)	6.24	23.8
	DPS	368	1.55 ± 0.18	12.67 (2.259 - 25.800)	65.20	11.6
	DPN	287	1.27 ± 0.15	8.83 (1.793 - 20.486)	46.81	8.1
	WHE	184	0.65 ± 0.09	6.02 (1.411 - 19.797)	9.56	5.5
Synergized Etofenprox	LAB	1037	0.81 ± 0.05	0.33 (0.193 - 0.614)	192.97	1.0
	AHN	427	1.21 ± 0.11	4.86 (3.075 - 8.270)	31.09	14.8
	AHC	100	1.23 ± 0.22	2.93 (1.484 - 6.075)	2.04	8.9
	DPS	159	0.72 ± 0.11	2.19 (0.098 - 3067.618)	35.45	6.7
	DPN	495	0.92 ± 0.07	1.94 (0.789 - 4.263)	85.60	5.9
Unsynergized Sumithrin	LAB	865	0.99 ± 0.06	1.23 (0.240 - 4.605)	702.77	1.0
	AHN	288	0.95 ± 0.11	6.51 (0.694 - 20.766)	58.77	5.3
	AHC	308	1.29 ± 0.14	12.84 (8.660 - 18.613)	14.15	10.4
	DPS	329	1.78 ± 0.22	11.77 (5.800 - 18.074)	22.92	9.5
	DPN	326	1.25 ± 0.13	9.62 (NA - NA)	1206.42	7.8

Active Ingredient	Site	n	Slope ± SE	LC$_{50}$ (95% CI)	χ^2	RR
Unsynergized Sumithrin	WHE	170	0.72 ± 0.11	16.53 (0.394 - 827.464)	29.31	13.4
Synergized Sumithrin	LAB	1042	0.92 ± 0.06	0.39 (0.232 - 0.784)	235.55	1.0
	AHN	392	1.10 ± 0.12	10.14 (4.246 - 34.927)	67.95	25.4
	AHC	122	2.25 ± 0.45	17.31 (10.857 - 29.033)	0.02	43.5
	DPN	477	1.31 ± 0.11	4.79 (2.379 - 9.267)	80.17	12.0
	WHE	174	1.39 ± 0.19	6.76 (4.296 - 10.692)	4.95	17.0
Unsynergized Deltamethrin	LAB	754	0.97 ± 0.06	0.005 (0.002 - 0.009)	186.81	1.0
	AHN	148	1.66 ± 0.27	1.025 (0.619 - 1.563)	3.63	219.8
	AHC	312	1.66 ± 0.20	0.897 (0.602 - 1.228)	2.42	192.4
	DPS	101	2.45 ± 0.52	0.334 (0.196 - 0.543)	0.03	71.7
	DPN	158	2.15 ± 0.38	0.796 (0.457 - 1.201)	3.98	170.7
	WHE	234	1.58 ± 0.21	0.318 (0.060 - 0.803)	30.89	68.1
Synergized Deltamethrin	LAB	1050	1.28 ± 0.07	0.004 (0.003 - 0.005)	130.65	1.0
	AHN	331	0.84 ± 0.09	0.208 (0.049 - 2.169)	92.47	59.0
	DPS	80	1.19 ± 0.25	0.028 (NA - NA)	16.18	8.0
	DPN	315	1.75 ± 0.18	0.066 (0.042 - 0.107)	24.93	18.9
	WHE	204	1.03 ± 0.13	0.0428 (0.013 - 0.162)	24.06	12.2
